# Corrigendum

**DOI:** 10.2471/BLT.15.110415

**Published:** 2015-04-01

In Volume 92, Issue 8, August 2014, page 565, the second sentence of the findings section of the abstract should read: “In 56% (62/111) of the samples, it exceeded the Bangladeshi threshold of 50 μg/l; the mean concentration being 54.5 μg/l (range: 0.1–193.1).”

